# Constrained State Estimation for Individual Localization in Wireless Body Sensor Networks

**DOI:** 10.3390/s141121195

**Published:** 2014-11-10

**Authors:** Xiaoxue Feng, Hichem Snoussi, Yan Liang, Lianmeng Jiao

**Affiliations:** 1 School of Automation, Northwestern Polytechnical University, Xi'an 710072, China; E-Mails: 15929443901@163.com (Y.L.); jiaolianmeng@163.com (L.J.); 2 Institute of Charles Delaunay, University of Technology of Troyes, Troyes 10000, France; E-Mail: hichem.snoussi@utt.fr

**Keywords:** constrained state estimation, ultra-wideband radio, individual localization, wireless body sensor networks

## Abstract

Wireless body sensor networks based on ultra-wideband radio have recently received much research attention due to its wide applications in health-care, security, sports and entertainment. Accurate localization is a fundamental problem to realize the development of effective location-aware applications above. In this paper the problem of constrained state estimation for individual localization in wireless body sensor networks is addressed. Priori knowledge about geometry among the on-body nodes as additional constraint is incorporated into the traditional filtering system. The analytical expression of state estimation with linear constraint to exploit the additional information is derived. Furthermore, for nonlinear constraint, first-order and second-order linearizations via Taylor series expansion are proposed to transform the nonlinear constraint to the linear case. Examples between the first-order and second-order nonlinear constrained filters based on interacting multiple model extended kalman filter (IMM-EKF) show that the second-order solution for higher order nonlinearity as present in this paper outperforms the first-order solution, and constrained IMM-EKF obtains superior estimation than IMM-EKF without constraint. Another brownian motion individual localization example also illustrates the effectiveness of constrained nonlinear iterative least square (NILS), which gets better filtering performance than NILS without constraint.

## 1. Introduction

In recent years, ultra-wide-band (UWB) technologies have drawn great interest in the wireless community [1]. The development of UWB has ushered in a new era in short-range wireless communications. Among various potential applications, one of the most promising applications is wireless body sensor networks (WBSNs) [2–4] based on UWB radio, which consist multiple low-cost, low data-rate and self-organized wireless sensors attached to, or implanted into, the human body, and have recently received a lot of research interests due to their wide applications in health-care, security, sports and entertainment. For location-aware applications requiring indoor positioning or motion capture features, global position information of the WBSNs or each individual sensor position are required [5,6].

In the WBSNs localization context, location estimators can be divided into two categories, non-probabilistic and probabilistic estimators. The typical algorithm of non-probabilistic estimator is weighted least squares (WLS) [2] which does not necessitate prior information. While probabilistic estimator consists in locating the mobile nodes based on probabilistic assumptions. The latter can be based on a priori statistical models for the observed measurements conditioned on the mobile positions, such as likelihood functions. Accordingly, these algorithms are usually more accurate than simple non-probabilistic estimators like WLS in the view of information fusion.

The probabilistic estimators can be classified into two categories, namely Bayesian and Non-Bayesian approaches. Non-Bayesian estimators assume that the mobile positions are treated as unknown deterministic parameters whereas mobile positions are defined as random variables with known prior distributions in Bayesian estimators. Kalman Filter (KF), as one of the most popular Bayesian estimator has been mainly adopted for tracking applications [7,8], for instance in vehicular or personal navigation applications. But in typical wireless tracking problems, due to the non-linearity of the measurement function, the Extended Kalman Filter (EKF) may be adopted instead, which consists in preserving the full KF formalism after linearizing locally the incriminated function around the predicted state. Both KF and EKF are well known and popular in the wireless body sensor networks [9–11], due to their simplicity and practicability for implementation. In the case of maneuvering targets, where the target switches between multiple states, the interactive multiple model (IMM) is employed. IMM uses a bank of filters processing in parallel, with each filter acting on a dynamic model.

Apart from the state filtering system, we might have information about a system that the filtering algorithm does not incorporate. For example, we may know that the states satisfy equality or inequality constraints. There are many examples of state-constrained systems in engineering applications. Some of these examples include, fault diagnosis [12], vision-based systems [13], target tracking [14,15], robotics [16], and navigation [17]. Recently, fixed-length links between on-body modes in WBSNs as additional constraints to improve the localization accuracy have been paid much attention. Mhedhbi *et al.* [18] adapts a centralized classical Multidimensional Scaling (MDS) for on-body MoCap applications and pose estimation. The authors introduce additional constraints relying on the prior knowledge of minimal and maximal feasible distances related to the body dimensions (and thus some kinds of geographical limitations). In [19] the centralized Maximum Likelihood estimator has been considered, introducing other constraints relying on the actual positions of on-body mobile nodes. In [20], the Constrained Distributed Weighted Multi-Dimensional Scaling (CDWMDS) algorithm is proposed for coarse WBAN motion capture, where nodes' locations are asynchronously estimated in a body-strapped Local Coordinate System (LCS), using information from their 1-hop neighbors. Fixed-length links (e.g., between the hand's wrist and the elbow) are also incorporated as geometric constraints, limiting the number of required on-line measurements, while still benefitting from a mesh topology. However, the above methods are all based non-Bayesian localization method.

In this paper, we investigate Bayesian localization method for UWB-based individual localization and aim at increasing the localization accuracy. Due to the harsh propagation environment in vicinity of the human body, the positioning accuracy can be degraded [21]. The major sources of this impairment are multi-path propagation and potential non-line-of-sight conditions. To overcome these problems, we investigate the improvement in the positioning accuracy by taking the a priori knowledge about geometry among the on-body nodes into account. For example, the distances between any two on-body nodes are fixed. From a practical point of view, the prior knowledge of the on-body nodes' distance can be obtained by letting the user deploy the nodes within a reasonably given area (e.g., drawn on a specific piece of clothes, typically on the torso). This information can be interpreted as additional reference measurements, which improves the localization accuracy in the view of information fusion. Our goal is how to introduce the fixed-length link constraints into the traditional Bayesian filtering algorithm.

In this paper, considering the priori knowledge about geometry among the on-body nodes, a novel constrained state estimation for individual localization is presented. Employing the Taylor series expansion of the nonlinear constraints and maintaining the first term and second term separately, IMM-EKF with first-order and second-order linearizations constraint are proposed respectively. Furthermore we discuss the two linearized methods influenced on the state estimation results through simulations. In this paper, our contribution is that we propose the constrained IMM-EKF algorithm for UWB based individual localization, exploit second-order nonlinear state constraints providing better approximation for higher order nonlinearities and demonstrate the effectiveness of the new method on an individual localization example, compared with the unstrained IMM-EKF. Note that, even the proposed constrained state estimation method is intended for Bayesian filtering algorithm, it can also be applied into the non-Bayesian algorithm, such as nonlinear iterative least square (NILS) [22].

The paper is organized as follows. Section 2 gives a brief formulation of individual localization problem. Section 3 presents the projection method for state estimation with linear constraint, and the first-order and second-order linearizations are proposed to extend the projection method to nonlinear cases. Section 4 applies the constrained state estimation into IMM-EKF for UWB based individual localization. Section 5 presents some simulation results to evaluate the algorithm. Finally, Section 6 closes with some conclusions and suggestions for future works.

## 2. Problem Formulation

**Figure 1.**
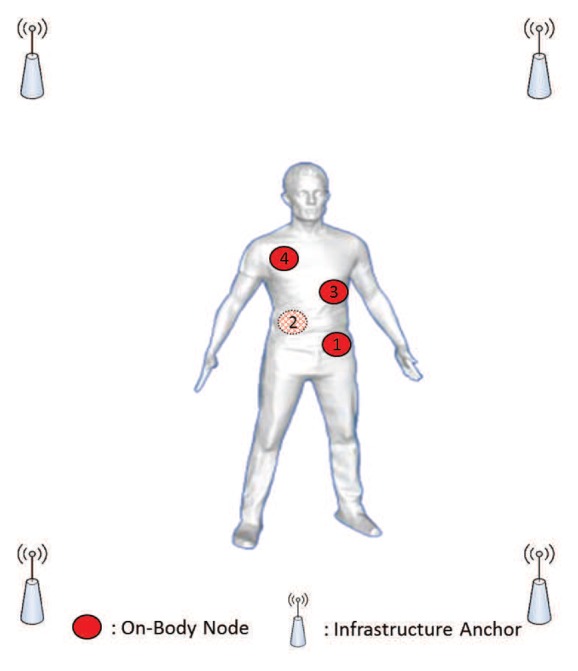
Typical WBSNs deployment scenario for individual localization.

A typical WBSNs indoor localization scenario is presented in Figure 1. Each WBSNs consists *m* + *n* nodes, where *n* is the number of mobile nodes to be located on the body (the red dots in Figure 1), and *m* is the number of anchors with known positions (the infrastructure anchors in Figure 1), where *m* should be equal or larger than 3. In the following, [*X*_1_(*k*), ⋯, *X_n_*(*k*)] is the vector of unknown 3-Dimensional coordinates at time *k* and [*X_n+_*_1_, ⋯, *X_n_*_+_*_m_*] is a vector of known and time-invariant positions of anchors.

We assume that anchors broadcast periodic beacon signals on a dedicated control channel, and that each WBSNs operates on a different channel frequency to avoid interference stemming from anchors and neighboring WBSNs. Moreover, we consider that each node is able to perform TDOA based (Time-Difference-Of-Arrival) range measurements [23] with respect to anchors within communication range and TOA based (Time-Of-Arrival) range measurements to other nodes associated to the same body area network. These distance measurements can be used together with the known anchor positions to localize the corresponding node relative to the anchors. Then the movement of the body is tracked by determining the nodes positions. Consider the following dynamic equation and measurement equation of the system given by

(1) X(k)=F(X(k−1))+n(k)

(2) Z(k)=G(X(k))+w(k)

where *F*(·) and *G*(·) are the known state transition function and measurement function, respectively. State vector *X*(*k*) includes the three-dimensional positions and velocities of each blind node to be positioned, *X*(*k*) = [*x*(*k*) *v_x_*(*k*) *y*(*k*) *v_y_*(*k*) *z*(*k*) *v_z_*(*k*)]. {*n*(*k*)} and {*w*(*k*)} are noise inputs. They are Gaussian distributions with zero mean and with covariance *Q*(*k*) and *R*(*k*). The two types of noise are mutually independent. Given the initial state vector and the associated covariance, the problem is to estimate the state vector of every time step by using corresponding measurement data.

Given all the available IR-UWB TOA measurements {*d̃_ij_*(*k*)}*_i,j_* at time *k*, given existing constraints related to the body geometry and the known locations of the infrastructure anchors, the problem considered in this paper consists in estimating the absolute positions of the carrying bodies in the global coordinate system, relying on their on-body nodes.

## 3. State Estimation with Constraint

### 3.1. State Estimation with Linear Constraint

Considering the same system defined in Equations (1) and (2), suppose there are some linear constraints in the state evolvement which are formulated using an equality formula as

(3) DX=δ

where *D* denotes a state constrained matrix. For simplification, time instant *k* is omitted. At this time, the filtering case is called a linear filtering problem with linear equality constraints. One method to deal with the equality linear constraints has been presented by Dan Simon [12,17]. It is a projection method in essence. It is an effective method and has a good performance. The detailed derivation is given in the following.

Let *X̃* denotes the modified state estimation, *W* denotes arbitrary symmetric positive definite matrix, *X̂* denotes the general state estimation before considering the condition of constraints. The estimation problem with state constraints translates into an optimization problem

(4) $$\{\begin{array}{l} \min J\left(\tilde{X}\right)={\left(\tilde{X}-\hat{X}\right)}^{T}W\left(\tilde{X}-\hat{X}\right)\\ \text{s}.\text{t}.D\tilde{X}=\delta \end{array}$$

Use the Lagrange multiplier method to solve the above equation. Firstly, we construct the formula

(5) $$J\left(\tilde{X},\lambda \right)={\left(\tilde{X}-\hat{X}\right)}^{T}W\left(\tilde{X}-\hat{X}\right)+2\lambda^{T}\left(D\tilde{X}-\delta \right)$$

Subsequently, calculate the partial derivatives and the results of each component are set zero, *i.e.*,

(6) $$\{\begin{array}{l} \frac{\partial J}{\partial X}=W\left(\tilde{X}-\hat{X}\right)+D^{T}\lambda =0\\ \frac{\partial J}{\partial \lambda }=D\tilde{X}-\delta =0 \end{array}$$

Thus we obtain

(7) $$\tilde{X}=\hat{X}-W^{-1}D^{T}{\left(DW^{-1}D^{T}\right)}^{-1}\left(D\hat{X}-\delta \right)$$

The method is called projection method via modifying state estimation. That is to say, the current state estimation is projected on the constrained subspace.

### 3.2. Sate Estimation with First-Order Linearization Nonlinear Constraint

For the nonlinear constraint *g*(*X*) = *d*, we can perform a Taylor series expansion of the constraint equation around the state prediction *X̂*^−^ to obtain

(8) $$g\left(X\right)\approx g\left({\hat{X}}^{-}\right)+g\prime \left({\hat{X}}^{-}\right)\left(X-{\hat{X}}^{-}\right)+1/2\sum_{i=1}^{s}e_{i}{\left(X-{\hat{X}}^{-}\right)}^{T}g^{\prime \prime }\left({\hat{X}}^{-}\right)\left(X-{\hat{X}}^{-}\right)$$

where *s* is the dimension of *g*(*X*), *e_i_* is the *i* natural basis vector in *R^s^*, and the entry in the *p*th row and *q*th column of the *n* × *n* matrix *g″*(*X*) is given by

(9) $${\left[g^{\prime \prime }\left(X\right)\right]}_{pq}=\frac{\partial^{2}g_{i}\left(X\right)}{\partial X_{p}\partial X_{q}}$$

Neglecting the second-order term gives

(10) $$g^{\prime }\left({\hat{X}}^{-}\right)X=d-g\left({\hat{X}}^{-}\right)+g^{\prime }\left({\hat{X}}^{-}\right){\hat{X}}^{-}$$

This equation is equivalent to the linear constraint if

(11) $$\{\begin{array}{l} D=g^{\prime }\left({\hat{X}}^{-}\right)\\ \delta =d-g\left({\hat{X}}^{-}\right)\_g\left({\hat{X}}^{-}\right){\hat{X}}^{-} \end{array}$$

Thus nonlinear constraints are linearized. Sometimes, though, we can do better than simple first-order linearization, as discussed in the following sections.

### 3.3. State Estimation with Second-Order Linearization Nonlinear Constraint

If we keep the second-order term in Taylor series expansion then the constrained estimation problem is second-order linearization nonlinear constraints. Then the constrained estimation problem can be approximately written as

(12) $$\tilde{X}=\underset{X}{\arg\min}{\left(X-\hat{X}\right)}^{T}W\left(X-\hat{X}\right)$$

such that

(13) $$X^{T}M_{i}X+2m_{i}^{T}X+\mu_{i}=0\left(i=1,\ldots ,s\right)$$

where *W* is a weighting matrix, and *M_i_*, *m_i_* and *μ_i_* can obtained from Equation (8). The optimization problem given in Equations (12) and (13) can be solved with a numerical method. A Lagrange multiplier method for solving this problem is given below [24–26].

(14) $$\hat{X}=G^{-1}V{\left(I+\lambda \sum^{T}\sum \right)}^{-1}e\left(\lambda \right)$$

(15) $$q\left(\lambda \right)=\sum_{i}\frac{e_{i}^{2}\left(\lambda \right)e_{i}^{2}}{{\left(1+\lambda \sigma_{i}^{2}\right)}^{2}}+2\sum_{i}\frac{e_{i}\left(\lambda \right)t_{j}}{1+\lambda \sigma_{i}^{2}}+m_{0}=0$$

where *G* is an upper right diagonal matrix resulting from the Cholesky factorization of *W* = *H^T^ H* as

(16) $$W=H^{T}H=G^{T}G$$

*V* is an orthonormal matrix, and Σ a diagonal matrix with its diagonal elements denoted by *σ_i_*, are obtained from the singular value decomposition (SVD) of the matrix *LG*^−1^ as

(17) $$LG^{-1}=U\sum V^{T}$$

where *U* is the other orthonormal matrix of the SVD and *L* results from the factorization *M* = *L^T^ L*, and

(18) $$e\left(\lambda \right)={\left[\ldots e_{i}\left(\lambda \right)\ldots \right]}^{T}=V^{T}{\left(G^{T}\right)}^{-1}\left(H^{T}z-\lambda m\right)$$

(19) $$t={\left[\ldots t_{i}\ldots \right]}^{T}=V^{T}{\left(G^{T}\right)}^{-1}m$$

As a nonlinear equation in λ, it is difficult to find a closed-form solution in general for the nonlinear equation *q*(*λ*) = 0 in Equation (15). Numerical root-finding algorithms, the Newton's method may be used instead. Consider the case where *W* = *G^T^G*, *z* = *GX* and *m* = 0, the constrained solution is given by

(20) $$\tilde{X}={\left(W+\lambda M\right)}^{-1}W\hat{X}$$

where the Lagrangian multiplier λ is obtained iteratively as below

(21) $$\lambda_{l+1}=\lambda_{l}-\frac{q\left(\lambda_{l}\right)}{\dot{q}\left(\lambda_{l}\right)}$$

with the corresponding *q*(*λ*) and *q̇*(*λ*) given by

(22) $$q\left(\lambda \right)=\sum_{i}\frac{e_{i}^{2}\sigma_{i}^{2}}{\left(1+\lambda \sigma_{i}^{2}\right)}+m_{0}=0$$

(23) $$\dot{q}\left(\lambda \right)=-2\sum_{i}\frac{e_{i}^{2}\sigma_{i}^{4}}{{\left(1+\lambda \sigma_{i}^{2}\right)}^{3}}$$

## 4. Constrained IMM-EKF for Individual Navigation

### 4.1. Unconstrained IMM-EKF for Individual Navigation

In order to solve non-linear tracking problems with behavior pattern of target changing with time, e.g., the measurement metric is based on IR-UWB TOA, interacting multiple model extended Kalman filter (IMM-EKF) is applied in this paper to perform individual localization. The IR-UWB TOA based measurement equation of node *i* in LOS condition is given below

(24) $$Z^{i}\left(k\right)=\left[\begin{array}{c} {\tilde{d}}_{i1}\left(X\left(k\right)\right)\\ {\tilde{d}}_{i2}\left(X\left(k\right)\right)\\ \vdots \\ {\tilde{d}}_{in+m-1}\left(X\left(k\right)\right) \end{array}\right]==\left[\begin{array}{c} {\left(X_{i}^{T}\left(k\right)X_{1}\left(k\right)\right)}^{\frac{1}{2}}+w_{i1}\left(k\right)\\ {\left(X_{i}^{T}\left(k\right)X_{2}\left(k\right)\right)}^{\frac{1}{2}}+w_{i2}\left(k\right)\\ \vdots \\ {\left(X_{i}^{T}\left(k\right)X_{n+m-1}\left(k\right)\right)}^{\frac{1}{2}}+w_{in+m-1}\left(k\right) \end{array}\right],j=1,2,\ldots ,n+m-1\text{and}j\neq i$$

where *d̃_ij_*(*X*(*k*)) denotes one range measurement available at time *k* between one on-body node *i* and a connected node *j*, *j* being another on-body node(belonging to the same WBSNs) or one infrastructure anchor. In case of IR-UWB, according to the IEEE 802.15.4a standard, the conditional TOA-based ranging error model is assumed to be similar for both of on-body and inter-body links. Measurement noise *w_ij_*(*k*) is a centered Gaussian random variable with a standard deviation *σ*.

The main steps in one cycle of IMM-EKF are given below [27]. For simplicity, the subscript *i* of node state *X_i_* is omitted.

Step 1 Calculate the mixing probabilities
  (25) $$\mu_{\alpha |\beta }\left(k|k\right)=\frac{p_{\alpha \beta }\mu_{\alpha }\left(k|k\right)}{\mu_{\beta }\left(k+1|k\right)}$$
  where the predicted mode probability *μ_β_*(*k* + 1|*k*) of mode *β* is computed by
  (26) $$\mu_{\beta }\left(k+1|k\right)=\sum_{\alpha =1}^{r}p_{\alpha \beta }\mu_{\alpha }\left(k|k\right)$$
  with *p_αβ_* being transition probability from mode *α* to mode *β* and *r* being the total number of probable model of state.
Step 2 Calculate the mixed initial state and covariance
  (27) $${\hat{X}}_{0\beta }\left(k|k\right)=\sum_{\alpha =1}^{r}\mu_{\alpha |\beta }\left(k|k\right){\hat{X}}_{\alpha }\left(k|k\right)$$
  (28) $${\hat{P}}_{0\beta }\left(k|k\right)=\sum_{\alpha =1}^{r}\mu_{\alpha |\beta }\left(k|k\right)\left\{{\hat{P}}_{\alpha }\left(k|k\right)+\left[{\hat{X}}_{\alpha }\left(k|k\right)-{\hat{X}}_{0\beta }\left(k|k\right)\right]{\left[•\right]}^{T}\right\}$$
Step 3 Perform mode-matched filtering and calculate the likelihood function Λ*_α_* corresponding to different model *α* via traditional EKF. When the EKF method is applied for solving non-linear tracking system, the main problem of EKF is to linearize locally the function around the predicted state. The linearized measurement equation *G_k_* satisfies
  (29) $$Z^{i}\left(k\right)≜G_{k}X\left(k\right)=\left[\begin{array}{c} {\tilde{d}}_{i1}\left(X\left(k\right)\right)\\ {\tilde{d}}_{i2}\left(X\left(k\right)\right)\\ \vdots \\ {\tilde{d}}_{in+m-1}\left(X\left(k\right)\right) \end{array}\right]=\left[\begin{array}{c} G_{k}^{1}X\left(k\right)\\ G_{k}^{2}X\left(k\right)\\ \ldots \\ G_{k}^{n+m-1}X\left(k\right) \end{array}\right],j=1,2,\ldots ,n+m-1\text{and}j\neq i$$
  where
  (30) $$G_{k}^{j}={\left[\begin{array}{llllll} \frac{\tilde{x}-x_{j}\left(k\right)}{D} & 0 & \frac{\tilde{y}-y_{j}\left(k\right)}{D} & 0 & \frac{\tilde{z}-z_{j}\left(k\right)}{D} & 0 \end{array}\right]}_{\tilde{X}=X_{i}\left(k|k-1\right)}$$
  (31) $$D={\left(X{\left(k|k-1\right)}^{T}X_{j}\left(k\right)\right)}^{\frac{1}{2}}$$
Step 4 Update model probability
  (32) $$\mu_{\beta }\left(k+1|k+1\right)=\frac{\mu_{\beta }\left(k+1|k\right)\Lambda_{\beta }}{\sum_{\alpha =1}^{r}\mu_{\beta }\left(k+1|k\right)\Lambda_{\alpha }}$$
Step 5 Combine model-conditioned estimates and covariances
  (33) $$X\left(k+1|k+1\right)=\sum_{\alpha =1}^{r}\mu_{\alpha }\left(k+1|k+1\right){\hat{X}}_{\alpha }\left(k+1|k+1\right)$$
  (34) $$\hat{P}\left(k+1|k+1\right)=\sum_{\alpha =1}^{r}\mu_{\alpha }\left(k+1|k+1\right)\left\{{\hat{P}}_{\alpha }\left(k+1|k+1\right)+\left[{\hat{X}}_{\alpha }\left(k+1|k+1\right)-\hat{X}\left(k+1|k+1\right)\right]{\left[•\right]}^{T}\right\}$$

### 4.2. Constrained IMM-EKF with First-Order Linearization Nonlinear Constraint (FC-IMM-EKF)

The additional constraints introduced in the individual localization system is that the distance between any two on-body nodes is fixed (*i.e.*, constant over time under body mobility) represented as follows. For simplification, time index *k* is omitted.

(35) $${\left(x_{i}-x_{j}\right)}^{2}+{\left(y_{i}-y_{j}\right)}^{2}+{\left(z_{i}-z_{j}\right)}^{2}={d_{ij}}^{2}$$

Obviously, the constraint is nonlinear. The deduced results of first-order linearization constraint in Equation (11) are given below.

(36) $$D={\left[\begin{array}{llllll} x-x_{j}\left(k\right) & 0 & x-x_{j}\left(k\right) & 0 & x-x_{j}\left(k\right) & 0 \end{array}\right]}_{X=X_{i}\left(k|k-1\right)}$$

(37) $$\delta ={d_{ij}}^{2}-X_{i}\left(k|k-1\right)X_{j}^{T}\left(k\right)+DX_{i}\left(k|k-1\right)$$

Thus the modified state estimation after first-order linearization constraint is

(38) $${\tilde{X}}_{i}\left(k|k\right)=X_{i}\left(k|k\right)-W^{-1}D^{T}{\left(DW^{-1}D^{T}\right)}^{-1}\left(DX_{i}\left(k|k\right)-\delta \right)$$

with *W* being an identity matrix or the inverse of estimation covariance *P*.

### 4.3. Constrained IMM-EKF with Second-Order Linearization Nonlinear Constraint (SC-IMM-EKF)

The most difficult problem of second-order linearization nonlinear constraint is to find the solution of λ. However, for the special constraint case mentioned in Equation (35), a closed-form solution of λ can be derived. Assume *W* = *I*_2_, *M* = *I*_2_, *m* = 0, and $m_{0}=-d_{ij}^{2}$. The constrained estimate in Equation (20) can be written below. For simplification, time index *k* is omitted.

(39) $$\tilde{X}-X_{j}={\left(W+\lambda M\right)}^{-1}W\left(\hat{X}-X_{j}\right)={\left(1+\lambda \right)}^{-1}\left(\hat{X}-X_{j}\right)$$

Substitute the above equation into the constraint equation in Equation (35)

(40) $${\left(\tilde{X}-X_{j}\right)}^{T}\left(\tilde{X}-X_{j}\right)={\left(\frac{\hat{X}-X_{j}}{1+\lambda }\right)}^{T}\left(\frac{\hat{X}-X_{j}}{1+\lambda }\right)=d_{ij}^{2}$$

The solution for λ is

(41) $$\lambda =\frac{\sqrt{{\left(\hat{X}-X_{j}\right)}^{T}\left(\hat{X}-X_{j}\right)}}{d_{ij}}-1$$

Taking the solution of λ back into Equation (39) gives the deduced results of second-order linearization constraint in Equations (12) and (13)

(42) $${\tilde{X}}_{i}\left(k|k\right)=X_{j}\left(k|k\right)+\frac{d_{ij}\left(X_{i}\left(k|k\right)-X_{j}\left(k\right)\right)}{\sqrt{{\left(X_{i}\left(k|k\right)-X_{j}\left(k\right)\right)}^{T}\left(X_{i}\left(k|k\right)-X_{j}\left(k\right)\right)}}$$

## 5. Simulation Results

In our evaluation framework, only an individual localization is considered. The body moves in a 20 *m* × 20 *m ×* 4 *m* 3D environment. The scene is surrounded by 4 infrastructure anchors, set at the corners. And there are 4 nodes placed on the body. The initial position of the 4 nodes are given in Table 1. And the measurement noise covariance is 0.09. The distance among the on-body nodes as additional constraints are given in Table 2 as below.

### 5.1. Simulation Case 1: Localize the CV Target Based on EKF

In the first simulation case, the individual moves at the constant speed of 0.4 *m*/*s* for an overall duration of 50 *s*. In other words, the model number *r* equals 1. The target state transition equation used in the CV tracking is

(43) $$X_{k+1}=\left[\begin{array}{llllll} 1 & T & 0 & 0 & 0 & 0\\ 0 & 1 & 0 & 0 & 0 & 0\\ 0 & 0 & 1 & T & 0 & 0\\ 0 & 0 & 0 & 1 & 0 & 0\\ 0 & 0 & 0 & 0 & 1 & T\\ 0 & 0 & 0 & 0 & 0 & 1 \end{array}\right]X_{k}+n_{k}$$

where process noise *n_k_* subject to Gaussian distribution, zero-mean with covariance *Q* = *diag*([0.004, 0.002, 0.003, 0.002, 0, 0]).

**Figure 2.**
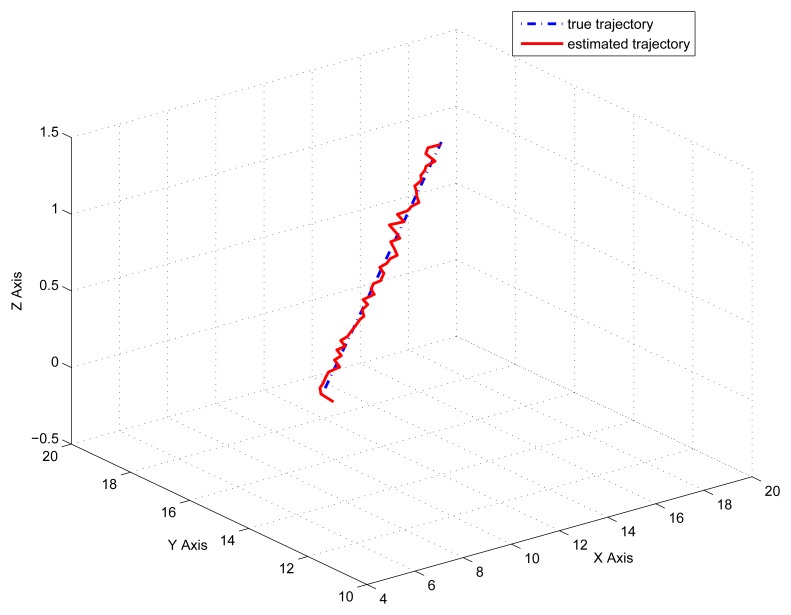
Estimation result of EKF.

**Figure 3.**
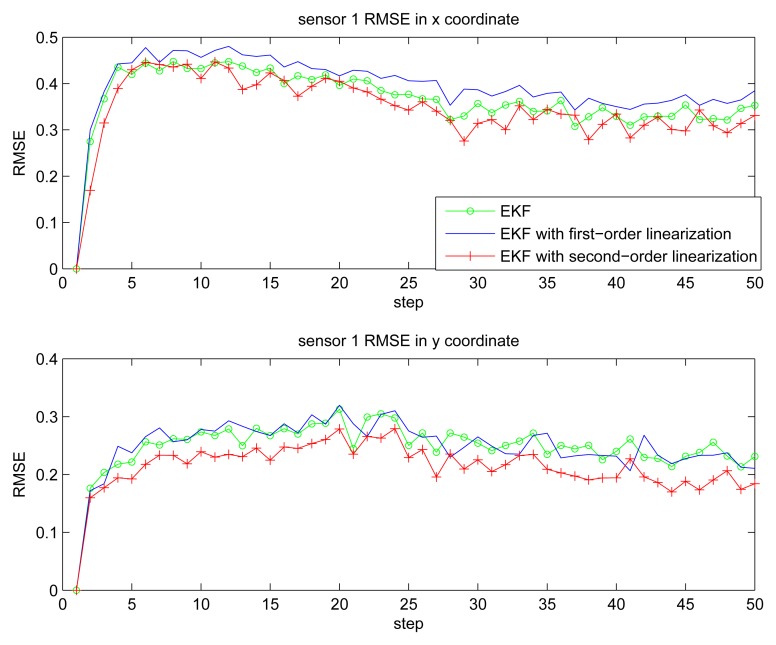
RMSE of sensor 1.

**Figure 4.**
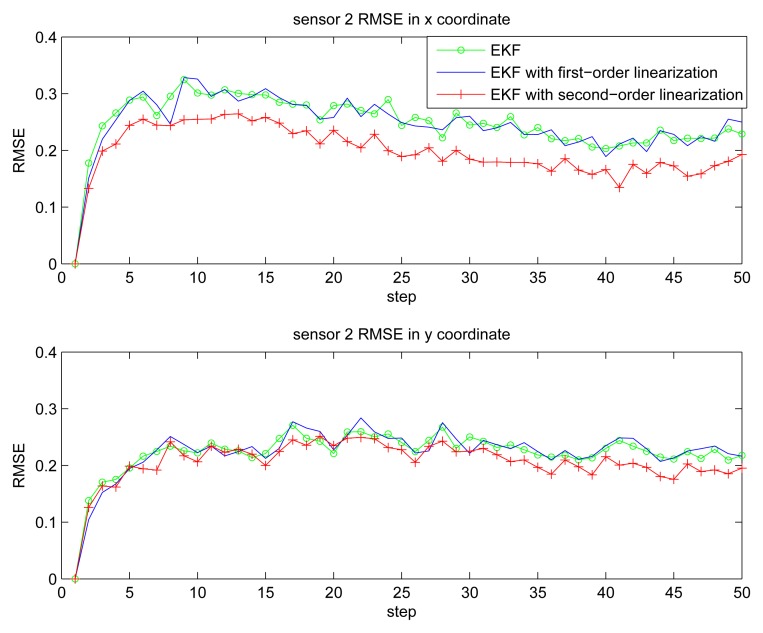
RMSE of sensor 2.

**Figure 5.**
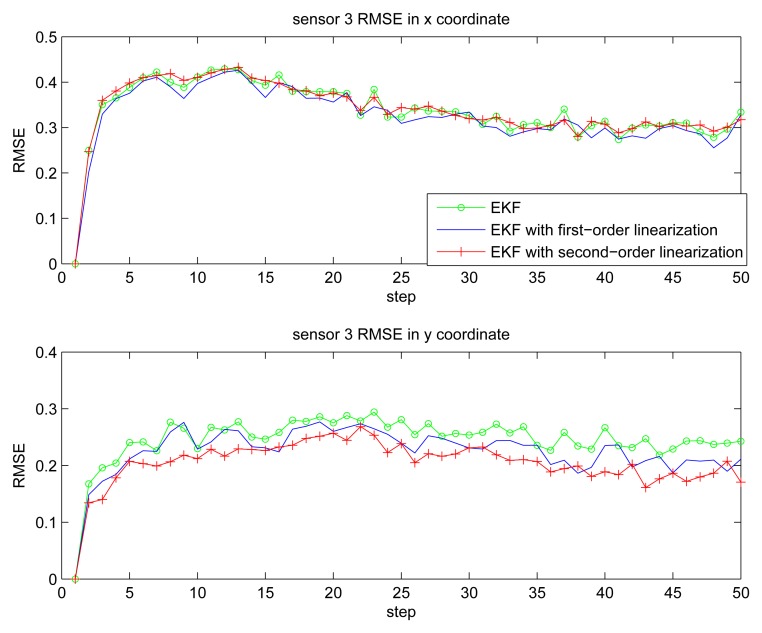
RMSE of sensor 3.

**Figure 6.**
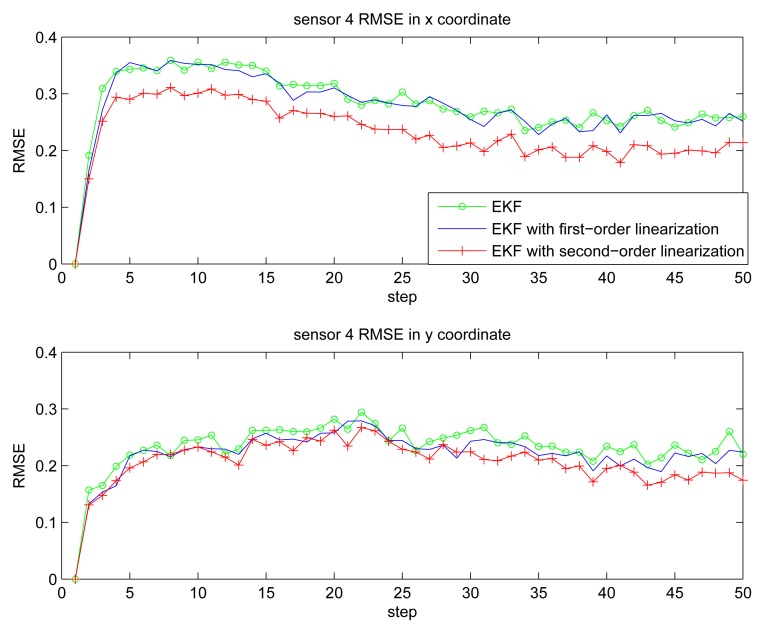
RMSE of sensor 4.

**Figure 7.**
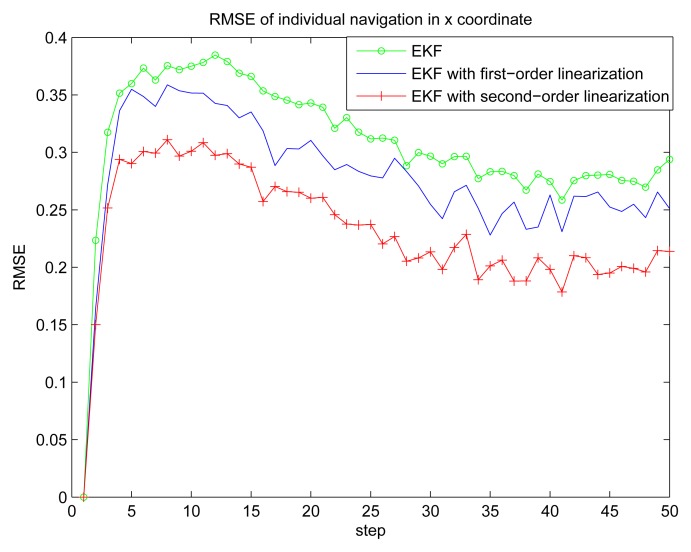
RMSE of individual localization in x coordinate.

**Figure 8.**
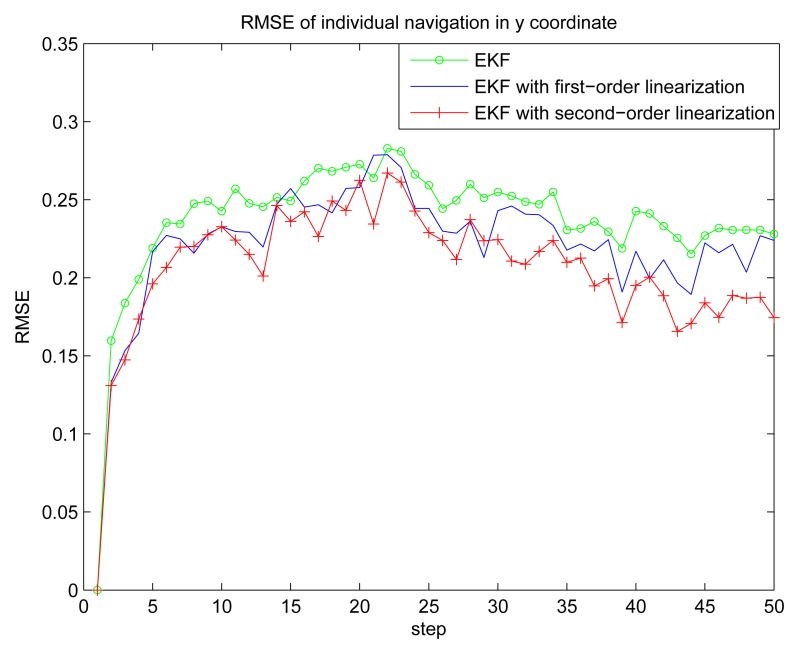
RMSE of individual localization in y coordinate.

Figure 2 gives the estimation result of EKF based individual localization, which shows the method of EKF based individual localization is effective. In the following figures, comparisons among EKF, EKF with the first-order nonlinear constraints (FC-EKF) and EKF with second-order nonlinear constraints (SC-EKF) of localization accuracy based on 100 Monte-Carlo runs are given. Figures 3, 4, 5 and 6 show estimation results of 4 on-body nodes and Figures 7–8 show estimation results of individual localization. It can be seen that the constrained EKF results in much more accurate estimates than the unconstrained EKF, which verifies the effectiveness of the proposed method. Also, it can be seen that the estimation results of FC-EKF is worse than SC-EKF. The reason of this phenomenon is mainly the state constraints is of high order nonlinearity. Thus we can conclude that for higher-order nonlinear constraints, the second-order solution as presented in this paper would outperform a first-order solution.

### 5.2. Simulation Case 2: Localize the Maneuvering (CV-CT-CA) Target Based on IMM-EKF

An IMM-EKF switching between a (nearly) constant velocity (CV), (nearly) constant acceleration (CA) models and a coordinated turn (CT) model is simulated here: (1) CV for the first 20 time intervals (2) CT for the 20-40 time intervals (3) CA for the final 20 time intervals. Target state transition equation used in the CA tracking is

(44) $$X_{k+1}=\left[\begin{array}{cccccc} 1 & T & T^{2}/2 & 0 & 0 & 0\\ 0 & 1 & T & 0 & 0 & 0\\ 0 & 0 & 1 & 0 & 0 & 0\\ 0 & 0 & 0 & 1 & T & T^{2}/2\\ 0 & 0 & 0 & 0 & 1 & T\\ 0 & 0 & 0 & 0 & 0 & 1 \end{array}\right]X_{k}+\left[\begin{array}{cc} T^{2}/2 & 0\\ T & 0\\ 1 & 0\\ 0 & T^{2}/2\\ 0 & T\\ 0 & 1 \end{array}\right]q_{1k}$$

where *q*_1_*_k_* is the variance of process noise *Q*_1_ = *diag*([0.001^2^, 0.001^2^]).

**Figure 9.**
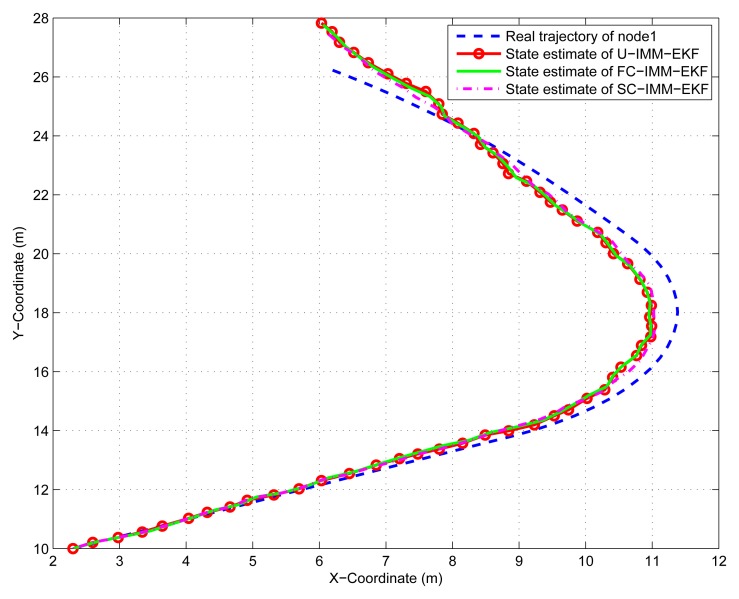
State estimation of node 1.

**Figure 10.**
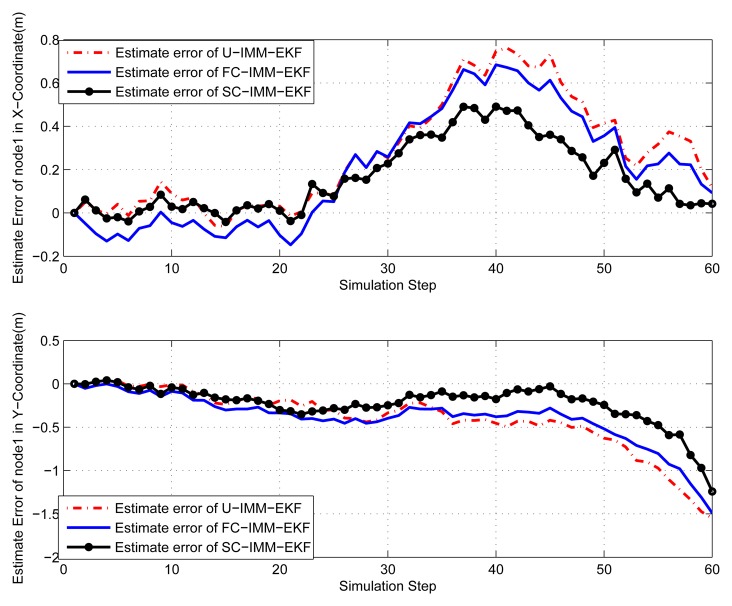
Estimate error of node 1.

**Figure 11.**
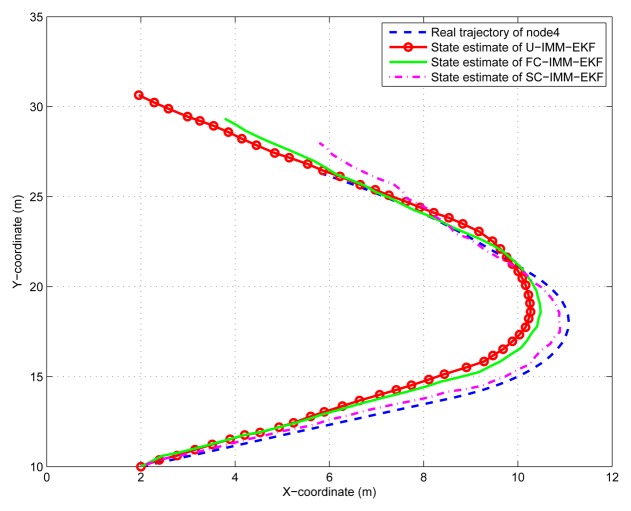
State estimation of node 4.

**Figure 12.**
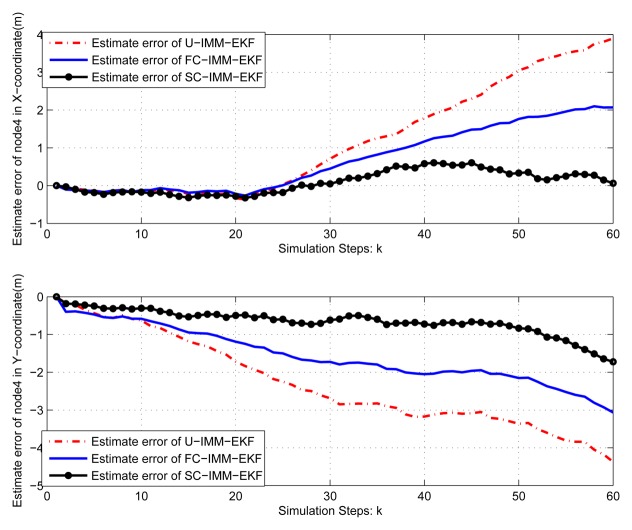
Estimate error of node 4.

Target state transition equation used in the CT tracking is

(45) $$X_{k+1}=\left[\begin{array}{cccccc} 1 & \sin\left(w*T\right)/w & 0 & 0 & -\left(1-\cos\left(w*T\right)\right)/w & 0\\ 0 & \cos\left(w*T\right) & 0 & 0 & -\sin\left(w*T\right) & 0\\ 0 & 0 & 0 & 0 & 0 & 0\\ 0 & \left(1-\cos\left(w*T\right)\right)/w & 0 & 1 & -\sin\left(w*T\right)/w & 0\\ 0 & \sin\left(w*T\right) & 0 & 0 & \cos\left(w*T\right) & 0\\ 0 & 0 & 0 & 0 & 0 & 0 \end{array}\right]X_{k}+\left[\begin{array}{cc} T^{2}/2 & 0\\ T & 0\\ 0 & 0\\ 0 & T^{2}/2\\ 0 & T\\ 0 & 0 \end{array}\right]q_{2k}$$

where *q*_2_*_k_* is the variance of process noise *Q*_2_ = *diag*([0.001^2^, 0.001^2^]), and the turn rate *ω* =0.08 *rad*/*s*. The IMM-EKF niters assume a mode transition probability matrix π = [0.8, 0.15, 0.05; 0.3, 0.4, 0.3; 0.05, 0.15, 0.8] and are initialized with the mode probability vector *μ* = [1/3, 1/3, 1/3]. In order to save space, only results of node 1 and node 4 are given here. The same conclusion can be obtained from the Figures 9, 10, 11 and 12, that is, the constrained IMM-EKF results in much more accurate estimates than the U-IMM-EKF, and the estimation results of SC-IMM-EKF is better than FC-IMM-EKF.

### 5.3. Simulation Case 3: Localize the Brownian Motion Target Based on NILS

**Figure 13.**
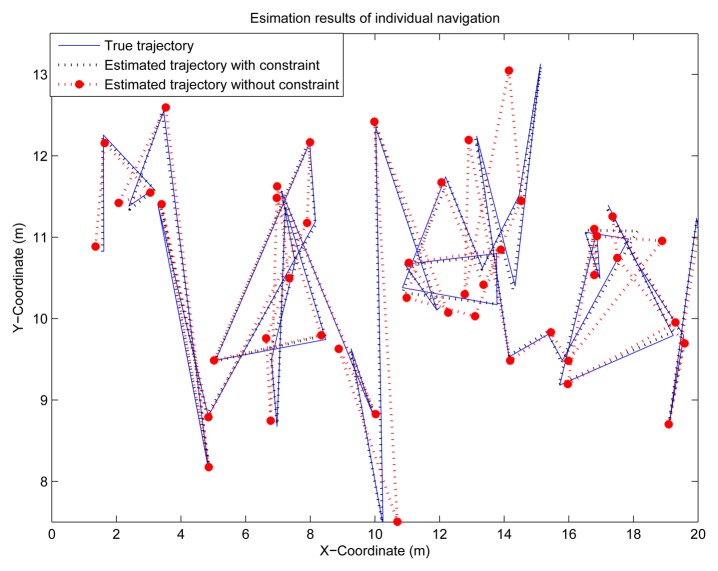
State estimation of individual localization.

**Figure 14.**
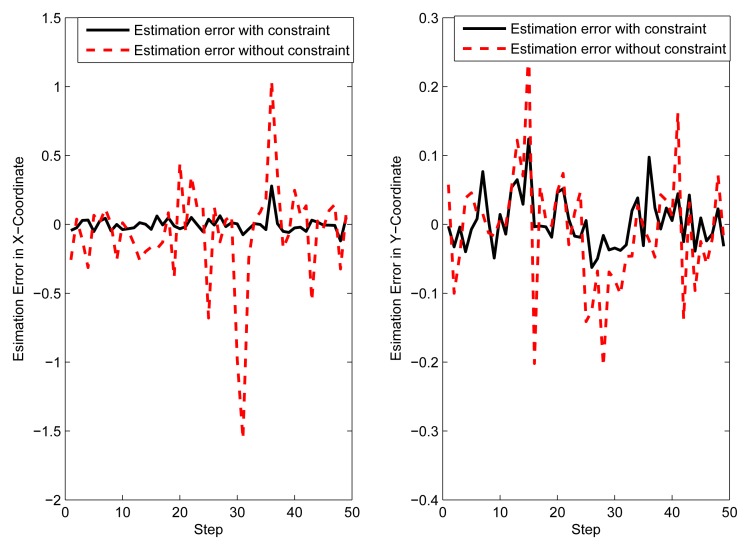
Estimate error of individual localization.

In order to prove that the proposed constrained state estimation can also be applied in non-Baysian methods, individual localization based on nonlinear iterative least square (NILS) [22] is simulated here. It can be seen from Figures 13 and 14, that the constrained NILS provides much more accurate estimates than the unconstrained NILS, which verifies the effectiveness of the proposed method.

## 6. Conclusion

In order to further improve the localization accuracy of the individual localization problem, priori knowledge about geometry among the on-body nodes as additional constraints are incorporated into the traditional IMM-extended Kalman filter. A novel IMM-EKF with nonlinear constraints for individual localization in WBSNs is presented. Employing the Taylor series expansion of the nonlinear constraints and maintaining the first term and second term separately, IMM-EKF with first-order and second-order linearizations constraint are proposed respectively. Simulation results on an individual localization example demonstrate that the proposed constrained IMM-EKF method gets better estimation performance than the unconstrained IMM-EKF provides. And it is exploited that second-order nonlinear state constraints providing better approximation than the first-order case for high order nonlinearity constraint. Besides, simulation about localizing a Brownian motion target based on NILS proves the effectiveness of the proposed method on non-Baysian algorithm.

In this paper, we utilize the Newton's method to find the numerical root in Equation (15). Our future works will include searching for more efficient root finding algorithm to solve the Lagrangian multiplier. Other directions of our future work will show more theoretical results related to convergence and accuracy for nonlinear constrained IMM-EKF.

## Figures and Tables

**Table 1.** Initial positions of the 4 nodes.

Index	1	2	3	4
*x* position (m)	2.3	2	2.2	2.1
*x* velocity (m/s)	0.3464	0.3464	0.3464	0.3464
*y* position (m)	10	10	10	10
*y* velocity (m/s)	0.2	0.2	0.2	0.2
*z* position (m)	0.8	0.7	0.8	1
*z* velocity (m/s)	0	0	0	0

**Table 2.** Distance constraint between each two nodes.

Index	1	2	3	4
1	**–**	0.3162m	0.1m	0.2828m
2	0.3162m	**–**	0.2236m	0.3162m
3	0.1m	0.2236m	**–**	0.2236m
4	0.2828m	0.3162m	0.2236m	**–**
